# Bispecific T Cell Engagers for the Treatment of Multiple Myeloma: Achievements and Challenges

**DOI:** 10.3390/cancers13122853

**Published:** 2021-06-08

**Authors:** Kinan Alhallak, Jennifer Sun, Amanda Jeske, Chaelee Park, Jessica Yavner, Hannah Bash, Berit Lubben, Ola Adebayo, Ayah Khaskiah, Abdel Kareem Azab

**Affiliations:** 1 Department of Radiation Oncology, Washington University in St. Louis School of Medicine, St. Louis, MO 63108, USA; kinanalhallak@wustl.edu (K.A.); jennifer.sun@wustl.edu (J.S.); ajeske@wustl.edu (A.J.); c.park@wustl.edu (C.P.); jyavner@wustl.edu (J.Y.); hannah.bash@wustl.edu (H.B.); berit.lubben@slu.edu (B.L.); olaadebayo@wustl.edu (O.A.); 2 Department of Biomedical Engineering, Washington University in St. Louis McKelvey School of Engineering, St. Louis, MO 63130, USA; 3 Faculty of Pharmacy, Nursing and Health Professions, Birzeit University, Birzeit 627, West Bank, Palestine; ayahkhaskiah@gmail.com

**Keywords:** bispecific T cell engagers, chimeric antigen receptor-T cells, multiple myeloma

## Abstract

**Simple Summary:**

Here we list the benefits and disadvantages of using bispecific T cell engagers (BTCEs) for the treatment of multiple myeloma (MM). We summarize the mechanism of action; the various targets used for BTCE therapy for MM such as BCMA, CD38, FcRH5, CD19, and CD138; and novel strategies used to circumvent the limitations of BTCE therapy.

**Abstract:**

MM is the second most common hematological malignancy and represents approximately 20% of deaths from hematopoietic cancers. The advent of novel agents has changed the therapeutic landscape of MM treatment; however, MM remains incurable. T cell-based immunotherapy such as BTCEs is a promising modality for the treatment of MM. This review article discusses the advancements and future directions of BTCE treatments for MM.

## 1. Introduction

Multiple myeloma (MM) is a neoplastic plasma cell dyscrasia that primarily arises in the bone marrow. It is the second most common hematological malignancy and represents approximately 20% of deaths from hematopoietic cancers [1]. Mainstay therapies for MM, such as corticosteroids, proteasome inhibitors, and immunomodulatory drugs, have shown significant clinical success and improved patient survival [2]. With the never-ending improvements of standard-of-care practices in MM, the current median survival has recently surpassed six years [3,4]. However, MM is notoriously incurable and patients who fall victim to this disease eventually relapse. Therefore, novel therapeutic strategies are warranted to improve the therapeutic landscape in MM.

T cell-based immunotherapy is solidifying itself as a major strategy for the treatment of MM. The concept of targeting T cells during the early stages of immunotherapy development was conceived following the observation of the T cell’s ability to eliminate cancers and harm normal tissue via graft-versus-leukemia and graft-versus-host disease, respectively [5]. This has led to extensive research in immunotherapies focused exclusively on T cells and ways to hone T cell-directed cytotoxicity on cancer cells while mitigating potential deleterious effects. Examples of T cell-based immunotherapy used for MM include immune checkpoint inhibitors, chimeric antigen receptor (CAR)-T cells, and bispecific T cell engagers (BTCEs) [6,7,8]. In this review, we provide a brief overview of BTCEs being investigated for the treatment of MM and address the general achievements and challenges of this emerging immunotherapy option.

## 2. Bispecific T Cell Engagers

### 2.1. Mechanism of Action

**Figure 1:**
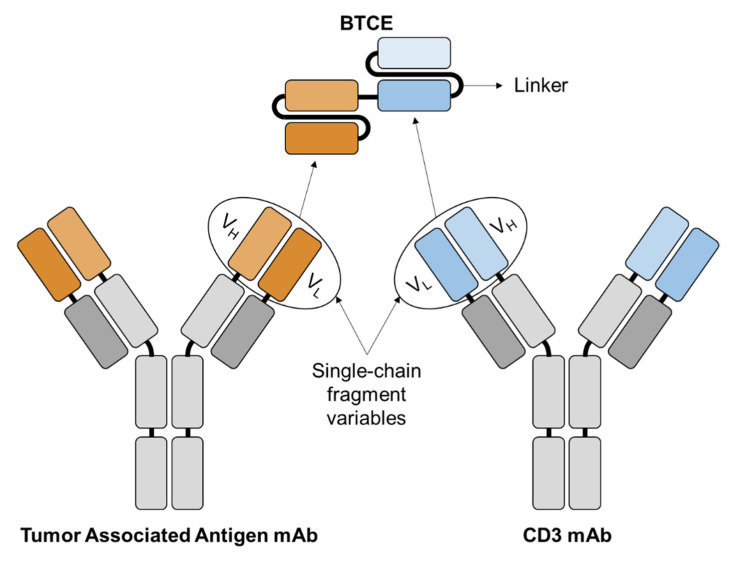
Creation of the BTCE using scFvs of two monoclonal antibodies (mAbs) linked together using a protein linker. The anti-tumor associated antigen scFv specifically recognizes the desired TAA on the tumor cell while the anti-CD3 scFv recognizes the CD3 molecule on the T cell. This enables a highly specific and bivalent system for T cell-based immunotherapy.

**Figure 2:**
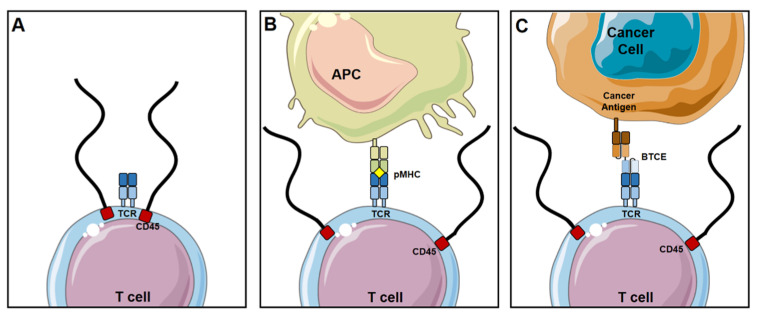
Molecular mechanism of BTCE-induced T cell activation. (**A**) The kinetic-segregation model proposes that the exclusion of CD45 is a prerequisite for T cell activation. (**B**) As the antigen presenting cell (APC) gets in proximity of the T cell, CD45 is subsequently excluded and the peptide major histocompatibility complex (pMHC) interacts with the T cell receptor (TCR) and enables activation. (**C**) For BTCE-induced T cell activation, the BTCE brings the tumor cell in proximity of the T cell to exclude CD45 from the close-contact zone and enable subsequent T cell activation.

BTCEs are a class of bispecific antibodies that are made up of two single chain variable fragments (scFvs) which are connected by a protein linker [9] as shown in Figure 1. The scFvs bind to MM and T cells by targeting the desired MM antigen and the CD3 subunit of the T cell receptor (TCR), respectively [10,11]. The first bispecific antibody that was produced and published on was in 1972 [12] and strategies for improving BTCE manufacturing have been always ongoing. Once the BTCE is bound to the target antigen and CD3, this subsequently leads to formation of an immune synapse, upregulation of T cell activation and granule expression, and polyclonal expansion of the T cells [13,14,15]. BTCE-induced T cell activation is potent, highly specific, independent of TCR specificity, does not need co-stimulation of CD28 and/or other co-stimulatory molecules, and does not require peptide antigen presentation for target cell lysis. BTCEs do not activate T cells by solely binding to the TCR due to their low affinity, the activation of T cells is only triggered upon concomitant binding of the TCR and target cell to the BTCE [16]. The general basis of how a BTCE activates a T cell is explained by the kinetic-segregation model [17] (Figure 2). CD45 is a transmembrane protein, constituted of a large extracellular domain and an intracellular phosphatase, the phosphatase domain of CD45 interacts with the TCR and dephosphorylates it, hence preventing its activation [18,19]. In a resting T cell, the net phosphorylation of the TCR is kept at a minimum due to dephosphorylation by CD45 [17], as shown in Figure 2A. Physiologically, when the T cell interacts with an antigen-presenting cell (APC), the TCR binds to the MHC with the antigen it is presenting, forming a close-contact zone immune-synapse. The close proximity of the T cells to the APC pushes away the extracellular domain of CD45, due to its large size (~30–50 nm) [20], which prevents the CD45 from interacting and dephosphorylating the TCR, and allows the activation of the T cell [21] (Figure 2B). BTCE-directed lytic synapses forms between T cells and target cells closely mimic those formed naturally through the TCR and MHC class peptide antigen interactions [22]; this is done by initiating an interaction between the T cell and target cell directly through cell specific antigens induced by the BTCE, as shown in Figure 2C. Once the BTCE is bound to the target antigen on the cancer cell and CD3 on T cells, the BTCE-induces formation of a close-contact zone immune-synapse that pushes the extracellular domain of CD45 away from the TCR, preventing its dephosphorylation and subsequently allowing T cell activation. The distance between the T cell and the other cells in the close-contact zones in the immune-synapse can be up to 300 nm for sufficient TCR stimulation [23]; nonetheless, smaller contact zones and size of target antigen leads to better activation and efficacy of BTCE [24].

### 2.2. Advantages

#### 2.2.1. High Potency and Efficacy

BTCEs prove highly promising as a therapy due to their high potency and efficacy. The high potency of BTCEs is reflected by the low concentrations (picomolar range or lower) and low effector: target ratios required to demonstrate significant, specific lysis of target cells [13,14,22,25]. In the presence of BTCEs, serial lysis of tumor cells by T cells has been demonstrated, allowing for a robust response [26]. BTCEs are able to stimulate the production of lytic synapses without the normal TCR/MHC antigen recognition mechanism [15,27]. The small size of the BTCEs (approximately 55 kDa and 11 nm in length) brings the T cell and target cell into close proximity necessary to form a synapse [28]. This mechanism explains the high efficacy of BTCEs, as they are able to overcome tumor immunosuppressive mechanisms to evade the immune system, such as downregulation of MHC antigen presentation and molecules for co-stimulation [15,28].

#### 2.2.2. Safety

In addition to their efficacy, BTCEs demonstrate suitable safety. BTCEs have demonstrated high selectivity for target antigens, with no signs of T cell activation in the absence of a target antigen [29]. Unlike CAR-T cells which are already activated ex vivo, with BTCEs, T cells only become activated when a target cell is also present and bound to the BTCE, minimizing potentially harmful cytokine secretion in the absence of the target tumor cell [9,30]. In a phase I clinical trial of Amgen’s BCMA/CD3 BTCE (NCT03836053) in relapsed and/or refractory MM patients, AMG 420 demonstrated rates of cytokine release syndrome lower than those found for CAR-T cells that are directed to the same target [8].

#### 2.2.3. Availability Off-the-Shelf

As a therapeutic, BTCEs are available in an “off-the-shelf” manner, ready for immediate treatment use [27,31]. They act through the activation of endogenous T cells; unlike CAR-T cells, no ex vivo manipulation of patient immune cells is necessary in order to achieve a direct interaction between T cells and target cells [26]. This decreases the need to determine patient tumor-specific antigens for manipulation of T cells ex vivo, which is particularly beneficial as some tumors may not have distinctive antigens for targeting [25,32].

#### 2.2.4. Lower Cost

Currently, a typical drug treatment regimen such as a combination of bortezomib, lenalidomide, and dexamethasone costs around $220,000 per year [33], daratumumab treatment alone costs $120,000 per year [33], while the newly approved belantamab mafodotin-blmf antibody-drug conjugate is estimated to cost $337,700 a year [34]. The FDA-approved BTCE for B-ALL, blinatumomab, sells for around $89,000 per course of therapy [35]; whereas, CAR-T cells carry a higher financial burden for MM patients with a cost of around $500,000 per treatment [36]. The low cost of producing BTCEs stems from the advanced technologies that are currently available for the production of antibodies. This might lead to further developments in perfecting the state-of-the-art techniques used for the assembly of BTCEs and hence decreasing the overall price of using BTCEs for MM treatment.

### 2.3. Challenges

#### 2.3.1. Poor Pharmacokinetic Profile

The small size of the traditional BTCE (approximately 55 kDa and 11 nm in length) confers its poor absorption, distribution, metabolism, and excretion properties [9]. Similar to other small proteins, the traditional BTCE is also systemically eliminated via nonspecific catabolism; whereas, monoclonal antibodies (~150 kDa) have prolonged distribution in the blood due to neonatal Fc receptor (FcRn)-regulated protection of the Fc receptor [37,38]. Blinatumomab and other BTCEs of the same format have a typical half-life of around 2 h; due to the very short half-life, BTCEs have to be continuously administered intravenously for a cycle of 28 days [39]. Circumventing the poor pharmacokinetic profile of the traditional BTCE is one of the main reasons that more efforts are transitioning to investigating BTCEs that contain an Fc receptor.

Methods to circumvent the poor pharmacokinetic profile of BTCEs include supplementing an Fc region onto the BTCE structure. AMG 701 is an example of this; Amgen included an Fc region onto the scFvs to be able to take advantage of the FcRn-regulated protection of the BTCE [37,38]. Another example of prolonging the pharmacokinetic profile of the BTCE is including a single chain domain antibody that binds to albumin. This also takes advantage of the FcRn-mediated serum half-life extension [37]. Additionally, anti-albumin technology has also been used to extend BTCE half-life by non-covalently binding to albumin which avoids low affinity Fc receptor binding [40].

#### 2.3.2. Laborious and Cumbersome to Produce

Generally, creating a particular monoclonal or bispecific antibody takes about six months [41]; this is due to the long and laborious process that is required to successfully create the BTCE of interest. The standard operating procedure for making a BTCE is first started by creating the desired DNA constructs using gene synthesis [12]. Phage display is used to develop the sequences of human variable fragments [42]. Once the preferred gene is isolated, assembled, and sequenced, restriction enzymes are introduced at both ends of the scFv gene to induce ligation of the gene and plasmid for subsequent cloning and plasmid construction [43]. The above process is repeated once more for the creation of the second scFv. Both scFvs are linked together using a short peptide that contain glycine and serine which are most commonly used for linkers [44]; this method is done by polymerase chain reaction. The resulting product is expressed in a bacterial or mammalian system such as Escherichia coli or Chinese hamster ovary cells, respectively, to achieve larger quantities of the BTCE [45,46]. Following propagation, the BTCE is reduced and refolded to create active molecules. Then the final product is achieved by purification via ion-exchange chromatography. Protein concentration and purity is finally assessed using Bradford assay and sodium dodecyl sulfate–polyacrylamide gel electrophoresis, respectively [43,46].

#### 2.3.3. Inability to Target Multiple Antigens

**Figure 3:**
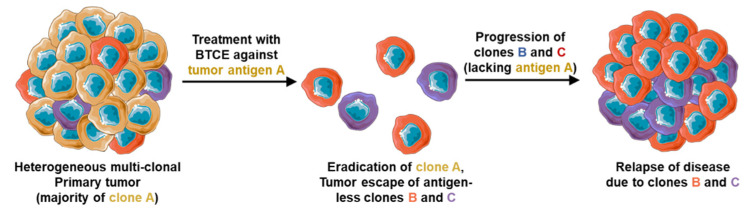
Mechanism of tumor escape and relapse after BTCE therapy targeting only one surface marker due to development of antigen-less clones.

Cancer is a multi-clonal disease, each clone can express different patterns of tumor antigens. Within the same patients the existence of several clones that may express different levels (or no levels) of tumor antigens expressed on the dominant clone was observed [47], which may significantly limit the efficacy of BTCEs targets one tumor antigen only. To further explain this phenomenon, we demonstrated the concept schematically in Figure 3. Assuming a multi-clonal tumor with three different each has high expression of different surface antigens A, B or C, with the clone expressing antigen A as the dominant clone. The estimative approach to treat this tumor would be an anti-antigen A BTCE, which may indeed eradicate the clone with high expression of antigen A, but leaving behind the other two clones B and C, which are antigen-less of A, to escape the treatment, proliferate and induce relapse of the disease. In addition, antigen loss or downregulation of specific surface antigens is a common mechanism observed in cancer cells treated with targeted therapy against the specific antigen [48]. For instance, patient treated with BCMA-targeted CAR-T cells, BCMA expression on MM cells was decreased significantly [49], which raises the need to create BTCEs with the ability to target multiple tumor antigens simultaneously to circumvent antigen-less tumor escape and patient relapse.

In addition to the incapability of targeting more than one surface antigen on the cancer cell, there is also a need to target additional antigens on the T cell, other than CD3. Targeting CD28, in addition to CD3, as co-stimulatory receptor was shown to significantly induce more profound and sustained activation and proliferation of T cells [50]. In CAR-T cells, persistence and antitumor lysis was significantly augmented when co-stimulatory molecules such as ICOS and 4-1BB were incorporated in the CAR [51]; therefore BTCEs also are in need of the ability to target co-stimulatory receptors to prevent exhaustion and increase the antitumor effects of T cells.

The development of TCEs that target multiple antigens is challenging due to the highly sophisticated nature of producing TCEs. Nabel and colleagues recently created a trispecific TCE targeting CD38, CD3, and CD28 for the treatment of MM [52]. They investigated the levels of cytokine secretions, T cell activation, and T cell-redirected MM lysis in vitro and in vivo induced by the trispecific TCE. However, several concerns remain following the production of the first trispecific TCE for MM such as safety and feasibility to be able to progress towards the potential of creating a more multivalent TCE for MM.

In addition, Harpoon Therapeutics is currently investigating a novel trispecific TCE (HPN217) targeting T cells, MM, and albumin using anti-CD3, anti-BCMA, and anti-albumin, respectively. As mentioned before, the rationale for including anti-albumin is to substantially increase half-life [40]. This creates a trispecific TCE that is only ~50 kDa which is a third of the size of a monoclonal antibody [40].

## 3. BTCEs for the Treatment of MM

The central tenet of making an efficacious BTCE is to be able to target the malignant cancer cells without harming normal tissue to reduce off-target toxicities. The optimal antigen target would have high and universal expression on the cancer cells but not on other normal cells [47]. MM tumors are multi-clonal, highly heterogeneous, and genetically unstable [53,54,55]. Due to the high mutational burden of MM and the multi-clonal nature of the tumors, selecting a single most preferred target is oftentimes challenging. We have listed below a list of the most pursued antigen targets for the treatment of MM using BTCEs:

### 3.1. BCMA

BCMA, B cell maturation antigen also known as CD269 and TNFRS17, mediates the survival and growth of B cells and plays a critical role in the maturation and differentiation of B cells to plasma cells [56]. Persistence and long-term survival of plasma cells are hindered when BCMA expression is knocked out of plasma cells [57]. Most importantly, malignant plasma cells express significantly higher levels of BCMA compared to their normal counterparts [58,59], which validates BCMA as a selective immunotherapeutic target for MM. There is a direct relationship of the overexpression and activation of BCMA as MM progresses, and BCMA is used a biomarker for MM due to its significant high expression [60,61]. In addition, BCMA is universally and preferentially expressed on plasma cells with little to no expression in other hematologic cells. The only exception are plasmacytoid dendritic cells which have been shown to help survival of MM in the bone marrow [62]. BCMA is a wildly popular drug target for antibody-drug conjugates, CAR-Ts, as well as BTCEs in MM [63].

Amgen’s BCMA/CD3 BTCE (AMG 420/BI 836909) is constructed with BCMA and CD3 scFvs connected by a protein linker. In the preclinical setting, hallmarks of T cell activation and cytolytic activity in MM cell lines and primary patient samples were observed [29]. The xenograft tumors and plasma cells in vivo and in cynomolgus monkeys, respectively, were eradicated. AMG 420 is currently in clinical trial to assess the maximum tolerated dose (MTD) in patients with relapsed and/or refractory MM (NCT03836053; Table 1). The maximum dose tested was 800 μg/day of continuous intravenous administration for four weeks which led to grade 3 adverse events; 400 μg/day was found to be the MTD for this study [64]. Serious adverse events were seen in half of the patient cohort which consisted of peripheral neuropathy and infections. Secondary outcomes included a response rate of 70% at the MTD and an overall response rate of 31%.

Another Amgen BTCE that is targeted to BCMA is AMG 701. The difference between Amgen’s two BTCEs is that AMG 701 has an extra Fc region to extend half-life. AMG 701 has been demonstrated to induce potent and specific MM cell lysis in vitro and in vivo [65]. In Amgen’s study, they found that the elimination half-life of AMG 701 is around 112 h in cynomolgus monkeys. This study has prompted initiation of phase I/II clinical study to investigate the pharmacokinetic, pharmacodynamics, and efficacy of AMG 701 (NCT03287908; Table 1).

An additional BCMA/CD3 BTCE, REGN5458, is currently being investigated by Regeneron Pharmaceuticals [66]. Structurally, REGN5458 contains an Fc region with BCMA Fab and CD3 Fab domains. Preclinical data conclude that REGN5458 induced T cell-mediated lysis of MM cell lines and primary plasma cells in vitro. Additionally, xenograft tumors were eliminated when dosed at 4 mg/kg intravenously for twice a week. A phase I/II clinical trial was subsequently initiated to investigate the dose-limiting toxicities of REGN5458 (NCT03761108; Table 1). All patients included in the clinical study exhibited MM progression after undergoing three or more prior lines of treatment. Two (50%) patients were minimal residual disease negative following a weekly administration of 6 mg of REGN5458, while five (71%) patients had treatment-emergent adverse events related to the study (NCT03761108).

TeneoBio has also created a BCMA BTCE called TNB-383B that eliminates MM cells in vitro and in mice with minimal toxicity [31]. TeneoBio has shown that TNB-383B has significantly lower cytokine release with sufficient anti-tumor efficacy compared to other BTCEs targeting BCMA. An advantage of TNB-383B is the use of fully human scFvs in the BTCE structure to avoid any unwanted immune response that can come from using mouse scFvs. TeneoBio has teamed up with AbbVie to conduct a clinical trial using TNB-383B to investigate the MTD and pharmacokinetic profile of the BTCE in patients with relapsed or refractory MM (NCT03933735; Table 1).

Harpoon Therapeutics’ trispecific HPN217 targeting CD3, BCMA, and albumin has been shown to induce cytotoxicity in vitro, demonstrating greater potent killing of MM cells with higher BCMA density per cell [67]. They have also shown CD69 and CD25 upregulation as well as cytokine secretion, which are all hallmark markers of T cell activation. MM cell lysis and pharmacokinetic profiles were shown in mice and cynomolgus monkeys, respectively. The extended half-life of HPN217 was around 85 h, whereas the normal BTCE half-life is around 2 h [39].

### 3.2. CD38

The CD38 receptor is a transmembrane glycoprotein that acts as an adhesion molecule and mediator for cell growth and calcium signaling for MM [68]. CD38 is highly expressed on the vast majority of MM cells, however it is also expressed (to lower extent) on various hematopoietic cells, including monocytes, B cells, T cells, and natural killer cells [69,70]. CD38 has served as a target for the treatment of MM for multiple treatment regimens and have shown promising results in the clinic for monoclonal antibodies, such as daratumumab, isatuximab, MOR202 [71,72], and CAR-T cells (NCT03464916), thus validating CD38 as a therapeutic target for MM.

Amgen’s CD38/CD3 BTCE (AMG 424) has been investigated in the preclinical setting [73], and Amgen is currently recruiting patients to begin a phase I/II clinical trial (NCT03445663; Table 1). AMG 424 deviates from the traditional BTCE structure that consists of only scFv fragments. An Fc region supports the base of AMG 424 with a CD38 fragment antigen binding (Fab) domain on one side and CD3 scFv on the other. AMG 424 induced MM cell killing in vitro and in vivo and depleted the targeted B cells in cynomolgus monkeys; B cells were the primary outcome in this study due to the technically challenging nature of tracking plasma cells in cynomolgus monkeys. However due to the ubiquitous expression of CD38 on normal tissue, the potential toxicities of AMG 424 were also assessed in the mentioned study. The authors concluded that the depletion of monocytes and T cells only occurred at significantly large doses (EC_50_ of 42 and 325 pmol/L, respectively) compared to the depletion of B cells which only had an EC_50_ of 8 pmol/L in peripheral blood mononuclear cells of cynomolgus monkeys [73]. This preclinical study has led to the initiation of a phase I/II clinical trial for the treatment of patients with relapsed and/or refractory MM (NCT03287908).

Another CD38/CD3 BTCE has been pushed to a phase I/II clinical trial by Ichnos Sciences (NCT03309111; GBR 1342; Table 1). According to Ichnos Sciences, the investigators delineate the structure of GBR 1342 to be very similar to that of AMG 424 [74]. The structure of GBR 1342 includes an Fc region with a CD38 scFv and CD3 Fab domain; whereas Amgen created AMG 424 with a CD38 Fab domain and CD3 scFv. GBR 1342 was shown to induce antitumor activity in vitro. The authors also monitored the depletion of T cells and monocytes in cynomolgus monkeys. They found that GBR 1342 depleted T cells and CD38-positive monocytes and observed a rebound of both cell types after approximately 48 h [75].

There is also a trispecific TCE targeting MM, a T cell co-stimulatory molecule, and the TCR by using anti-CD38, anti-CD28, and anti-CD3 [52]. The rationale for targeting CD28 is to enable enhanced and persistent T cell activation. The trispecific TCE enables cytolysis of MM and activation of T cells in vitro and in vivo. Additionally, Nabel’s group investigated the TCE in primates and found that the MTD varied based on administration. Intravenous administration showed an MTD of 30–75 ug/kg whereas for subcutaneous, MTD was greater than 100 ug/kg; this is most likely due to the greater serum antibody levels in the blood following intravenous injection.

### 3.3. FcRH5

FcRH5, also known as CD307, FcRL5, and IRTA2, is an immunoregulatory cell surface molecule that is expressed only on B cells and remains on their surface as they mature to plasma cells, unlike major B-cell markers such as CD19, CD20, and CD22, which are lost in plasma cells [76]. As an immunotherapeutic target, FcRH5 is highly attractive due to its consistent expression on different developmental stages of B cells and the ability to utilize FcRH5 as a general target for other B cell malignancies [77,78]. FcRH5 are always expressed on plasma cells; whereas other specific mature B cell markers are downregulated [76]. FcRH5 mRNA is additionally overexpressed in MM compared to other hematopoietic cells. FcRH5 is a universal and novel target and is being pursued for treatment regimens such as CAR-T cells [79].

Genentech created a BTCE with two Fab domains (one targeting FcRH5 and the other targeting CD3) and an Fc portion at the base of the bispecific IgG [80]. The proof-of-concept of Genentech’s FcRH5/CD3 BTCE has been extensively investigated [24,80]. Preclinically, the FcRH5/CD3 BTCE induced T cell activation in vitro concurring with the kinetic-segregation model, and the authors investigated the ability of the BTCE to induce T cell activation and killing as the targeted epitope location is distal, central, or proximal to the cell membrane [24]; they found that the membrane-proximal epitope produced a more efficient T cell synapse and enhanced killing of MM. FcRH5/CD3 BTCE was also shown to redirect T cells to lyse MM patient samples, a MM cell line, and plasma cells in vitro, in vivo, and in cynomolgus monkeys respectively [24]. In addition, Genentech recently optimized their FcRH5/CD3 BTCE to enable negligible antibody-dependent cell-mediated cytoxicity and investigated whether or not this would impair its ability to induce T cell activation and T cell-redirected MM cell lysis [80]. This BTCE will be translated to a phase I clinical trial to primarily determine the adverse events that occur during and after administration of the FcRH5/CD3 BTCE in MM patients (NCT03275103; Table 1).

### 3.4. CD19

CD19 is a cell surface marker that acts as a coreceptor in antigen receptor-mediated activation of B cells and enhances intracellular signaling [81]. Normal plasma cells express CD19, whereas generally CD19 is not present on the surface of MM [82]. CD19 has been only shown to be expressed on MM in rare occasions [83]. However, a certain population of MM expresses very low levels of CD19 and is known to have an aggressive stem-like phenotype [82,84].

Blinatumomab, a CD19/CD3 BTCE, has been approved for the treatment of B-cell acute lymphoblastic leukemia (B-ALL) [85,86]. Blinatumomab has been proposed to target this aggressive subset of MM. Yet, there are currently no published studies that investigate blinatumomab for MM preclinically, and the only clinical trial that is studying the feasibility and safety of blinatumomab for the treatment of MM has been terminated recently (NCT03173430; Table 1).

### 3.5. CD138

CD138 or syndecan-1 is a canonical cell marker that is highly expressed and very abundant on MM and plasma cells. CD138 has been shown to increase tumor progression and survival and induces angiogenesis, cytoskeletal formation, adhesion, and signaling [87]. It has also been shown to interact with cytokines, chemokines, and growth factors to exert molecular roles in tumorigenesis. The gold standard marker to detect MM is the use of CD138 due to the very high presence of the marker on MM [54]; however, CD138 can be shed which can regulate function and stability [88]. CD138 is universally expressed on MM cells; however, different perturbations to MM cells can decrease expression such as hypoxia which could be the reason for failure of many CD138-targeted therapies [89].

A CD138 BTCE has been made to combat MM cells with the targeted surface marker. This specific BTCE actually includes an Fc portion to engage natural killer cells as well as T cells [90]. This particular aspect of including an Fc region enables increased half-life (which was not shown in this study [90]) and engagement of natural killer cells to induce an even greater immune response against MM. They found that the BTCE bound to natural killer, T cells, and MM cells to form a complex that induced MM cell killing. The CD138 BTCE was able to upregulate CD69 and CD25 expression and activate CD4 and CD8 T cells. T cell-mediated MM cell lysis was observed using fluorescent microscopy and was able to induce anti-tumor efficacy in vitro and in vivo.

### 3.6. Novel TCE Strategies

Recently, nanoparticles, particularly liposomes, were used as a surrogate to bispecific antibodies for the engagement of T cells [91]. Nanoparticle-based T cell engager (nanoBTCE) were developed simply, reproducibly, and quickly using chemical conjugation of two monocloncal antibodies, the first against tumor-antigen and the second against CD3 on the T cells to the surface of the liposomes. The nanoBTCE has a size of approximately 130 nm, and once the nanoBTCE is bound to the target antigen on the cancer cell and CD3 on the T cells, the nanoBTCEs induced formation of a close-contact zone immune-synapse that allows T cell activation, and tumor lysis in vitro and in vivo [91]. In addition, the nanoBTCEs circumvented the poor pharmacokinetic profile of the classic BTCEs, with a half-life of 60 h in the blood in vivo. Most importantly, due to the highly heterogeneous nature, targeting one marker was shown to create antigen-less tumors and relapse for the MM patient, preclinical [91] and in clinical [49,92,93,94,95,96] settings. Therefore, nanoparticle-based multi-specific T cell engager (nanoMuTCE) were developed to target three most abundant cell surface antigens on MM; BCMA, CS1 (SLAMF7), and CD38, each of these markers are individually present on MM cells; however, expression differs from patient to patient [97]. The production of the nanoMuTCEs was reported to be simple, using chemical conjugation of four monocloncal antibodies to the surface of the liposomes, three against the tumor-antigens (BCMA, CS1, and CD38) and one against CD3 on the T cells, similar to the preparation of the nanoBTCEs. The nanoMuTCEs mediated more T cell activation and MM cell lysis in vitro and in vivo, compared to nanoBTCEs targeting one tumor antigen at a time. Single-antigen-targeted nanoBTCEs induced antigen-less tumor escape due to the elimination of MM cell clones only expressing the target antigen; whereas, nanoMuTCEs eliminated all MM cell clones and did not create any antigen-less MM clones [91]. Nanomaterials used for development of TCEs for MM and other cancers should be investigated further.

### 3.7. Toxicities

Compared to CAR-T cell therapy, BTCE therapy faces similar adverse effects but generally are much more manageable [26]. Cytokine release syndrome is common but seen in low grade [98,99]; Neurotoxicity has also been reported, in Amgen’s AMG420 [64] and more severely in Pfizer’s Elranatamab [100].

In addition, one major concern for targeting B cell markers is the potential toxicity due to depletion of B cells. This low B cell repertoire may inhibit the ability to produce a neutralizing IgG response, putting patients in an immunodeficient state in danger of serious infections. A CD19-targeted CAR-T cell therapy against diffuse large B cell lymphoma reported a substantial increase of infection occurrence in patients [101]; the CD19/CD3 BTCE Blinatumomab induced prolonged B cell aplasia [102]. While limited clinical trial data is available for plasma cell targeting BTCEs in MM, previous types of related therapy point to the possibility for similar B cell toxicities.

## 4. Conclusions

The development of TCEs for the treatment of MM is rapidly growing. There have been significant progress, clinically and preclinically, for the use of TCEs to activate T cells in the patient and eliminate MM. High potency and efficacy, safety, availability off-the-shelf, and low cost are all advantages of TCEs compared to other T cell immunotherapies. On the other hand, TCEs are still challenged by poor pharmacokinetic profile, laborious production, and inability to target multiple antigens. The use of biotechnology such as nanomaterials has the potential to circumvent limitations while maintaining or improving the current advantages. Future directions for development of TCEs for MM should include: (1) advancing the investigation of CS1 (SLAMF7) as a TCE target in vivo and in clinical trials based on promising results recently [91,103]; (2) focusing on development of trispecific TCEs to overcome antigen-less tumor relapse and treatment resistance; (3) expanding TCE technology to engage non-conventional T cells such as NK- and γδ T cells [104,105]; (4) interrogating TCE combination therapy with current therapies such as autologous stem cell transplant, daratumumab, and/or checkpoint inhibitors; (5) exploring novel formats such as nanomaterial, camelid nanobody, tandem diabody, dual-affinity retargeting antibody [106,107,108]. Taken together, TCE immunotherapy has immense potential and can be further improved as an efficient treatment for the clearance of MM and other cancers.

## Figures and Tables

**Table 1:** BTCE Clinical Trials in Multiple Myeloma.

BiTE Name	BiTE Target	Phase	Status	Clinical Trial Number	Completion Date
Blinatumomab	CD19	I	Terminated	NCT03173430	2019
AMG 424	CD38	I/II	Recruiting	NCT03445663	2022
GBR 1342	CD38	I/II	Recruiting	NCT03309111	2021
BFCR4350A	FcRH5	I	Recruiting	NCT03275103	2022
AMG 420	BCMA	I	Completed/ Active	NCT02514239/ NCT03836053	2020/ 2025
AMG 701	BCMA	I/II	Recruiting	NCT03287908	2025
CC-93269	BCMA	I	Recruiting	NCT03486067	2026
Elranatamab (PF-06863135)	BCMA	I/II	Recruiting/ Active	NCT03269136/ NCT04649359	2023
REGN5458	BCMA	I/II	Recruiting	NCT03761108	2022
TNB-383B	BCMA	I	Recruiting	NCT03933735	2021
